# Search for lepton-flavour-violating decays of Higgs-like bosons

**DOI:** 10.1140/epjc/s10052-018-6386-8

**Published:** 2018-12-12

**Authors:** R. Aaij, C. Abellán Beteta, B. Adeva, M. Adinolfi, C. A. Aidala, Z. Ajaltouni, S. Akar, P. Albicocco, J. Albrecht, F. Alessio, M. Alexander, A. Alfonso Albero, G. Alkhazov, P. Alvarez Cartelle, A. A. Alves, S. Amato, S. Amerio, Y. Amhis, L. An, L. Anderlini, G. Andreassi, M. Andreotti, J. E. Andrews, R. B. Appleby, F. Archilli, P. d’Argent, J. Arnau Romeu, A. Artamonov, M. Artuso, K. Arzymatov, E. Aslanides, M. Atzeni, B. Audurier, S. Bachmann, J. J. Back, S. Baker, V. Balagura, W. Baldini, A. Baranov, R. J. Barlow, S. Barsuk, W. Barter, F. Baryshnikov, V. Batozskaya, B. Batsukh, V. Battista, A. Bay, J. Beddow, F. Bedeschi, I. Bediaga, A. Beiter, L. J. Bel, S. Belin, N. Beliy, V. Bellee, N. Belloli, K. Belous, I. Belyaev, E. Ben-Haim, G. Bencivenni, S. Benson, S. Beranek, A. Berezhnoy, R. Bernet, D. Berninghoff, E. Bertholet, A. Bertolin, C. Betancourt, F. Betti, M. O. Bettler, M. van Beuzekom, Ia. Bezshyiko, S. Bhasin, J. Bhom, S. Bifani, P. Billoir, A. Birnkraut, A. Bizzeti, M. Bjørn, M. P. Blago, T. Blake, F. Blanc, S. Blusk, D. Bobulska, V. Bocci, O. Boente Garcia, T. Boettcher, A. Bondar, N. Bondar, S. Borghi, M. Borisyak, M. Borsato, F. Bossu, M. Boubdir, T. J. V. Bowcock, C. Bozzi, S. Braun, M. Brodski, J. Brodzicka, A. Brossa Gonzalo, D. Brundu, E. Buchanan, A. Buonaura, C. Burr, A. Bursche, J. Buytaert, W. Byczynski, S. Cadeddu, H. Cai, R. Calabrese, R. Calladine, M. Calvi, M. Calvo Gomez, A. Camboni, P. Campana, D. H. Campora Perez, L. Capriotti, A. Carbone, G. Carboni, R. Cardinale, A. Cardini, P. Carniti, L. Carson, K. Carvalho Akiba, G. Casse, L. Cassina, M. Cattaneo, G. Cavallero, R. Cenci, D. Chamont, M. G. Chapman, M. Charles, Ph. Charpentier, G. Chatzikonstantinidis, M. Chefdeville, V. Chekalina, C. Chen, S. Chen, S.-G. Chitic, V. Chobanova, M. Chrzaszcz, A. Chubykin, P. Ciambrone, X. Cid Vidal, G. Ciezarek, P. E. L. Clarke, M. Clemencic, H. V. Cliff, J. Closier, V. Coco, J. A. B. Coelho, J. Cogan, E. Cogneras, L. Cojocariu, P. Collins, T. Colombo, A. Comerma-Montells, A. Contu, G. Coombs, S. Coquereau, G. Corti, M. Corvo, C. M. Costa Sobral, B. Couturier, G. A. Cowan, D. C. Craik, A. Crocombe, M. Cruz Torres, R. Currie, C. D’Ambrosio, F. Da Cunha Marinho, C. L. Da Silva, E. Dall’Occo, J. Dalseno, A. Danilina, A. Davis, O. De Aguiar Francisco, K. De Bruyn, S. De Capua, M. De Cian, J. M. De Miranda, L. De Paula, M. De Serio, P. De Simone, C. T. Dean, D. Decamp, L. Del Buono, B. Delaney, H.-P. Dembinski, M. Demmer, A. Dendek, D. Derkach, O. Deschamps, F. Desse, F. Dettori, B. Dey, A. Di Canto, P. Di Nezza, S. Didenko, H. Dijkstra, F. Dordei, M. Dorigo, A. Dosil Suárez, L. Douglas, A. Dovbnya, K. Dreimanis, L. Dufour, G. Dujany, P. Durante, J. M. Durham, D. Dutta, R. Dzhelyadin, M. Dziewiecki, A. Dziurda, A. Dzyuba, S. Easo, U. Egede, V. Egorychev, S. Eidelman, S. Eisenhardt, U. Eitschberger, R. Ekelhof, L. Eklund, S. Ely, A. Ene, S. Escher, S. Esen, T. Evans, A. Falabella, N. Farley, S. Farry, D. Fazzini, L. Federici, P. Fernandez Declara, A. Fernandez Prieto, F. Ferrari, L. Ferreira Lopes, F. Ferreira Rodrigues, M. Ferro-Luzzi, S. Filippov, R. A. Fini, M. Fiorini, M. Firlej, C. Fitzpatrick, T. Fiutowski, F. Fleuret, M. Fontana, F. Fontanelli, R. Forty, V. Franco Lima, M. Frank, C. Frei, J. Fu, W. Funk, C. Färber, M. Féo Pereira Rivello Carvalho, E. Gabriel, A. Gallas Torreira, D. Galli, S. Gallorini, S. Gambetta, Y. Gan, M. Gandelman, P. Gandini, Y. Gao, L. M. Garcia Martin, B. Garcia Plana, J. García Pardiñas, J. Garra Tico, L. Garrido, D. Gascon, C. Gaspar, L. Gavardi, G. Gazzoni, D. Gerick, E. Gersabeck, M. Gersabeck, T. Gershon, D. Gerstel, Ph. Ghez, S. Gianì, V. Gibson, O. G. Girard, L. Giubega, K. Gizdov, V. V. Gligorov, D. Golubkov, A. Golutvin, A. Gomes, I. V. Gorelov, C. Gotti, E. Govorkova, J. P. Grabowski, R. Graciani Diaz, L. A. Granado Cardoso, E. Graugés, E. Graverini, G. Graziani, A. Grecu, R. Greim, P. Griffith, L. Grillo, L. Gruber, B. R. Gruberg Cazon, O. Grünberg, C. Gu, E. Gushchin, Yu. Guz, T. Gys, C. Göbel, T. Hadavizadeh, C. Hadjivasiliou, G. Haefeli, C. Haen, S. C. Haines, B. Hamilton, X. Han, T. H. Hancock, S. Hansmann-Menzemer, N. Harnew, S. T. Harnew, T. Harrison, C. Hasse, M. Hatch, J. He, M. Hecker, K. Heinicke, A. Heister, K. Hennessy, L. Henry, E. van Herwijnen, M. Heß, A. Hicheur, R. Hidalgo Charman, D. Hill, M. Hilton, P. H. Hopchev, W. Hu, W. Huang, Z. C. Huard, W. Hulsbergen, T. Humair, M. Hushchyn, D. Hutchcroft, D. Hynds, P. Ibis, M. Idzik, P. Ilten, K. Ivshin, R. Jacobsson, J. Jalocha, E. Jans, A. Jawahery, F. Jiang, M. John, D. Johnson, C. R. Jones, C. Joram, B. Jost, N. Jurik, S. Kandybei, M. Karacson, J. M. Kariuki, S. Karodia, N. Kazeev, M. Kecke, F. Keizer, M. Kelsey, M. Kenzie, T. Ketel, E. Khairullin, B. Khanji, C. Khurewathanakul, K. E. Kim, T. Kirn, S. Klaver, K. Klimaszewski, T. Klimkovich, S. Koliiev, M. Kolpin, R. Kopecna, P. Koppenburg, I. Kostiuk, S. Kotriakhova, M. Kozeiha, L. Kravchuk, M. Kreps, F. Kress, P. Krokovny, W. Krupa, W. Krzemien, W. Kucewicz, M. Kucharczyk, V. Kudryavtsev, A. K. Kuonen, T. Kvaratskheliya, D. Lacarrere, G. Lafferty, A. Lai, D. Lancierini, G. Lanfranchi, C. Langenbruch, T. Latham, C. Lazzeroni, R. Le Gac, A. Leflat, J. Lefrançois, R. Lefèvre, F. Lemaitre, O. Leroy, T. Lesiak, B. Leverington, P.-R. Li, T. Li, Z. Li, X. Liang, T. Likhomanenko, R. Lindner, F. Lionetto, V. Lisovskyi, X. Liu, D. Loh, A. Loi, I. Longstaff, J. H. Lopes, G. H. Lovell, D. Lucchesi, M. Lucio Martinez, A. Lupato, E. Luppi, O. Lupton, A. Lusiani, X. Lyu, F. Machefert, F. Maciuc, V. Macko, P. Mackowiak, S. Maddrell-Mander, O. Maev, K. Maguire, D. Maisuzenko, M. W. Majewski, S. Malde, B. Malecki, A. Malinin, T. Maltsev, G. Manca, G. Mancinelli, D. Marangotto, J. Maratas, J. F. Marchand, U. Marconi, C. Marin Benito, M. Marinangeli, P. Marino, J. Marks, P. J. Marshall, G. Martellotti, M. Martin, M. Martinelli, D. Martinez Santos, F. Martinez Vidal, A. Massafferri, M. Materok, R. Matev, A. Mathad, Z. Mathe, C. Matteuzzi, A. Mauri, E. Maurice, B. Maurin, A. Mazurov, M. McCann, A. McNab, R. McNulty, J. V. Mead, B. Meadows, C. Meaux, F. Meier, N. Meinert, D. Melnychuk, M. Merk, A. Merli, E. Michielin, D. A. Milanes, E. Millard, M.-N. Minard, L. Minzoni, D. S. Mitzel, A. Mogini, J. Molina Rodriguez, T. Mombächer, I. A. Monroy, S. Monteil, M. Morandin, G. Morello, M. J. Morello, O. Morgunova, J. Moron, A. B. Morris, R. Mountain, F. Muheim, M. Mulder, C. H. Murphy, D. Murray, A. Mödden, D. Müller, J. Müller, K. Müller, V. Müller, P. Naik, T. Nakada, R. Nandakumar, A. Nandi, T. Nanut, I. Nasteva, M. Needham, N. Neri, S. Neubert, N. Neufeld, M. Neuner, T. D. Nguyen, C. Nguyen-Mau, S. Nieswand, R. Niet, N. Nikitin, A. Nogay, N. S. Nolte, D. P. O’Hanlon, A. Oblakowska-Mucha, V. Obraztsov, S. Ogilvy, R. Oldeman, C. J. G. Onderwater, A. Ossowska, J. M. Otalora Goicochea, P. Owen, A. Oyanguren, P. R. Pais, T. Pajero, A. Palano, M. Palutan, G. Panshin, A. Papanestis, M. Pappagallo, L. L. Pappalardo, W. Parker, C. Parkes, G. Passaleva, A. Pastore, M. Patel, C. Patrignani, A. Pearce, A. Pellegrino, G. Penso, M. Pepe Altarelli, S. Perazzini, D. Pereima, P. Perret, L. Pescatore, K. Petridis, A. Petrolini, A. Petrov, S. Petrucci, M. Petruzzo, B. Pietrzyk, G. Pietrzyk, M. Pikies, M. Pili, D. Pinci, J. Pinzino, F. Pisani, A. Piucci, V. Placinta, S. Playfer, J. Plews, M. Plo Casasus, F. Polci, M. Poli Lener, A. Poluektov, N. Polukhina, I. Polyakov, E. Polycarpo, G. J. Pomery, S. Ponce, A. Popov, D. Popov, S. Poslavskii, C. Potterat, E. Price, J. Prisciandaro, C. Prouve, V. Pugatch, A. Puig Navarro, H. Pullen, G. Punzi, W. Qian, J. Qin, R. Quagliani, B. Quintana, B. Rachwal, J. H. Rademacker, M. Rama, M. Ramos Pernas, M. S. Rangel, F. Ratnikov, G. Raven, M. Ravonel Salzgeber, M. Reboud, F. Redi, S. Reichert, A. C. dos Reis, F. Reiss, C. Remon Alepuz, Z. Ren, V. Renaudin, S. Ricciardi, S. Richards, K. Rinnert, P. Robbe, A. Robert, A. B. Rodrigues, E. Rodrigues, J. A. Rodriguez Lopez, M. Roehrken, S. Roiser, A. Rollings, V. Romanovskiy, A. Romero Vidal, M. Rotondo, M. S. Rudolph, T. Ruf, J. Ruiz Vidal, J. J. Saborido Silva, N. Sagidova, B. Saitta, V. Salustino Guimaraes, C. Sanchez Gras, C. Sanchez Mayordomo, B. Sanmartin Sedes, R. Santacesaria, C. Santamarina Rios, M. Santimaria, E. Santovetti, G. Sarpis, A. Sarti, C. Satriano, A. Satta, M. Saur, D. Savrina, S. Schael, M. Schellenberg, M. Schiller, H. Schindler, M. Schmelling, T. Schmelzer, B. Schmidt, O. Schneider, A. Schopper, H. F. Schreiner, M. Schubiger, M. H. Schune, R. Schwemmer, B. Sciascia, A. Sciubba, A. Semennikov, E. S. Sepulveda, A. Sergi, N. Serra, J. Serrano, L. Sestini, A. Seuthe, P. Seyfert, M. Shapkin, Y. Shcheglov, T. Shears, L. Shekhtman, V. Shevchenko, E. Shmanin, B. G. Siddi, R. Silva Coutinho, L. Silva de Oliveira, G. Simi, S. Simone, N. Skidmore, T. Skwarnicki, M. W. Slater, J. G. Smeaton, E. Smith, I. T. Smith, M. Smith, M. Soares, l. Soares Lavra, M. D. Sokoloff, F. J. P. Soler, B. Souza De Paula, B. Spaan, E. Spadaro Norella, P. Spradlin, F. Stagni, M. Stahl, S. Stahl, P. Stefko, S. Stefkova, O. Steinkamp, S. Stemmle, O. Stenyakin, M. Stepanova, H. Stevens, A. Stocchi, S. Stone, B. Storaci, S. Stracka, M. E. Stramaglia, M. Straticiuc, U. Straumann, S. Strokov, J. Sun, L. Sun, K. Swientek, T. Szumlak, M. Szymanski, S. T’Jampens, Z. Tang, A. Tayduganov, T. Tekampe, G. Tellarini, F. Teubert, E. Thomas, J. van Tilburg, M. J. Tilley, V. Tisserand, M. Tobin, S. Tolk, L. Tomassetti, D. Tonelli, D. Y. Tou, R. Tourinho Jadallah Aoude, E. Tournefier, M. Traill, M. T. Tran, A. Trisovic, A. Tsaregorodtsev, G Tuci, A. Tully, N. Tuning, A. Ukleja, A. Usachov, A. Ustyuzhanin, U. Uwer, A. Vagner, V. Vagnoni, A. Valassi, S. Valat, G. Valenti, R. Vazquez Gomez, P. Vazquez Regueiro, S. Vecchi, M. van Veghel, J. J. Velthuis, M. Veltri, G. Veneziano, A. Venkateswaran, T. A. Verlage, M. Vernet, M. Veronesi, N. V. Veronika, M. Vesterinen, J. V. Viana Barbosa, D. Vieira, M. Vieites Diaz, H. Viemann, X. Vilasis-Cardona, A. Vitkovskiy, M. Vitti, V. Volkov, A. Vollhardt, D. Vom Bruch, B. Voneki, A. Vorobyev, V. Vorobyev, J. A. de Vries, C. Vázquez Sierra, R. Waldi, J. Walsh, J. Wang, M. Wang, Y. Wang, Z. Wang, D. R. Ward, H. M. Wark, N. K. Watson, D. Websdale, A. Weiden, C. Weisser, M. Whitehead, J. Wicht, G. Wilkinson, M. Wilkinson, I. Williams, M. R. J. Williams, M. Williams, T. Williams, F. F. Wilson, J. Wimberley, M. Winn, J. Wishahi, W. Wislicki, M. Witek, G. Wormser, S. A. Wotton, K. Wyllie, D. Xiao, Y. Xie, A. Xu, M. Xu, Q. Xu, Z. Xu, Z. Xu, Z. Yang, Z. Yang, Y. Yao, L. E. Yeomans, H. Yin, J. Yu, X. Yuan, O. Yushchenko, K. A. Zarebski, M. Zavertyaev, D. Zhang, L. Zhang, W. C. Zhang, Y. Zhang, A. Zhelezov, Y. Zheng, X. Zhu, V. Zhukov, J. B. Zonneveld, S. Zucchelli

**Affiliations:** 1 Centro Brasileiro de Pesquisas Físicas (CBPF), Rio de Janeiro, Brazil; 2 Universidade Federal do Rio de Janeiro (UFRJ), Rio de Janeiro, Brazil; 3 Center for High Energy Physics, Tsinghua University, Beijing, China; 4 Univ. Grenoble Alpes, Univ. Savoie Mont-Blanc, CNRS/IN2P3-LAPP, Annecy, France; 5 Clermont Université, Université Blaise Pascal, CNRS/IN2P3, LPC, Clermont-Ferrand, France; 6 Aix-Marseille Univ, CNRS/IN2P3, CPPM, Marseille, France; 7 LAL, Univ. Paris-Sud, CNRS/IN2P3, Université Paris-Saclay, Orsay, France; 8 LPNHE, Sorbonne Université, Paris Diderot, Sorbonne Paris Cité, CNRS/IN2P3, Paris, France; 9 I. Physikalisches Institut, RWTH Aachen University, Aachen, Germany; 10 Fakultät Physik, Technische Universität Dortmund, Dortmund, Germany; 11 Max-Planck-Institut für Kernphysik (MPIK), Heidelberg, Germany; 12 Physikalisches Institut, Ruprecht-Karls-Universität Heidelberg, Heidelberg, Germany; 13 School of Physics, University College Dublin, Dublin, Ireland; 14 INFN Sezione di Bari, Bari, Italy; 15 INFN Sezione di Bologna, Bologna, Italy; 16 INFN Sezione di Ferrara, Ferrara, Italy; 17 INFN Sezione di Firenze, Florence, Italy; 18 INFN Laboratori Nazionali di Frascati, Frascati, Italy; 19 INFN Sezione di Genova, Genoa, Italy; 20 INFN, Sezione di Milano-Bicocca, Milan, Italy; 21 INFN Sezione di Milano, Milan, Italy; 22 INFN Sezione di Cagliari, Monserrato, Italy; 23 INFN Sezione di Padova, Padua, Italy; 24 INFN Sezione di Pisa, Pisa, Italy; 25 INFN Sezione di Roma Tor Vergata, Rome, Italy; 26 INFN Sezione di Roma La Sapienza, Rome, Italy; 27 Nikhef National Institute for Subatomic Physics, Amsterdam, The Netherlands; 28 Nikhef National Institute for Subatomic Physics and VU University Amsterdam, Amsterdam, The Netherlands; 29 Henryk Niewodniczanski Institute of Nuclear Physics Polish Academy of Sciences, Kraków, Poland; 30 Faculty of Physics and Applied Computer Science, AGH-University of Science and Technology, Kraków, Poland; 31 National Center for Nuclear Research (NCBJ), Warsaw, Poland; 32 Horia Hulubei National Institute of Physics and Nuclear Engineering, Bucharest-Magurele, Romania; 33 Petersburg Nuclear Physics Institute (PNPI), Gatchina, Russia; 34 Institute of Theoretical and Experimental Physics (ITEP), Moscow, Russia; 35 Institute of Nuclear Physics, Moscow State University (SINP MSU), Moscow, Russia; 36 Institute for Nuclear Research of the Russian Academy of Sciences (INR RAS), Moscow, Russia; 37 Yandex School of Data Analysis, Moscow, Russia; 38 Budker Institute of Nuclear Physics (SB RAS), Novosibirsk, Russia; 39 Institute for High Energy Physics (IHEP), Protvino, Russia; 40 ICCUB, Universitat de Barcelona, Barcelona, Spain; 41 Instituto Galego de Física de Altas Enerxías (IGFAE), Universidade de Santiago de Compostela, Santiago de Compostela, Spain; 42 European Organization for Nuclear Research (CERN), Geneva, Switzerland; 43 Institute of Physics, Ecole Polytechnique Fédérale de Lausanne (EPFL), Lausanne, Switzerland; 44 Physik-Institut, Universität Zürich, Zurich, Switzerland; 45 NSC Kharkiv Institute of Physics and Technology (NSC KIPT), Kharkiv, Ukraine; 46 Institute for Nuclear Research of the National Academy of Sciences (KINR), Kyiv, Ukraine; 47 University of Birmingham, Birmingham, UK; 48 H.H. Wills Physics Laboratory, University of Bristol, Bristol, UK; 49 Cavendish Laboratory, University of Cambridge, Cambridge, UK; 50 Department of Physics, University of Warwick, Coventry, UK; 51 STFC Rutherford Appleton Laboratory, Didcot, UK; 52 School of Physics and Astronomy, University of Edinburgh, Edinburgh, UK; 53 School of Physics and Astronomy, University of Glasgow, Glasgow, UK; 54 Oliver Lodge Laboratory, University of Liverpool, Liverpool, UK; 55 Imperial College London, London, UK; 56 School of Physics and Astronomy, University of Manchester, Manchester, UK; 57 Department of Physics, University of Oxford, Oxford, UK; 58 Massachusetts Institute of Technology, Cambridge, MA USA; 59 University of Cincinnati, Cincinnati, OH USA; 60 University of Maryland, College Park, MD USA; 61 Syracuse University, Syracuse, NY USA; 62 Pontifícia Universidade Católica do Rio de Janeiro (PUC-Rio), Rio de Janeiro, Brazil; 63 University of Chinese Academy of Sciences, Beijing, China; 64 School of Physics and Technology, Wuhan University, Wuhan, China; 65 Institute of Particle Physics, Central China Normal University, Wuhan, Hubei China; 66 Departamento de Fisica, Universidad Nacional de Colombia, Bogotá, Colombia; 67 Institut für Physik, Universität Rostock, Rostock, Germany; 68 Van Swinderen Institute, University of Groningen, Groningen, The Netherlands; 69 National Research Centre Kurchatov Institute, Moscow, Russia; 70 National University of Science and Technology “MISIS”, Moscow, Russia; 71 National Research Tomsk Polytechnic University, Tomsk, Russia; 72 Instituto de Fisica Corpuscular, Centro Mixto Universidad de Valencia-CSIC, Valencia, Spain; 73 University of Michigan, Ann Arbor, USA; 74 Los Alamos National Laboratory (LANL), Los Alamos, USA

## Abstract

A search is presented for a Higgs-like boson with mass in the range 45 to 195$\,{{\mathrm {GeV/}}c^2}$ decaying into a muon and a tau lepton. The dataset consists of proton-proton interactions at a centre-of-mass energy of 8$~{\mathrm {TeV}}$, collected by the LHCb experiment, corresponding to an integrated luminosity of 2$\,\text{ fb }^{-1}$. The tau leptons are reconstructed in both leptonic and hadronic decay channels. An upper limit on the production cross-section multiplied by the branching fraction at 95% confidence level is set and ranges from 22$\,{\mathrm {pb}}$ for a boson mass of 45$\,{{\mathrm {GeV/}}c^2}$ to 4$\,{\mathrm {pb}}$ for a mass of 195$\,{{\mathrm {GeV/}}c^2}$.

## Introduction

Decays mediated by charged-lepton flavour-violating (CLFV) processes are forbidden in the Standard Model (SM). Their observation would be a clear sign for physics beyond the SM. Such processes are predicted by several theoretical models [1–8], in particular those based on an effective theory with relaxed renormalisability requirements [9], supersymmetric models [10–14], composite Higgs models [15, 16], Randall–Sundrum models [17, 18], and non-abelian flavour symmetry models [19]. Nonetheless, no evidence for CLFV effects has been reported to date.

The LEP experiments set stringent limits on the CLFV decay of the $$Z $$ boson [20–23]. In the presence of CLFV couplings, the decays to $$e ^\pm $$ $$\mu ^\mp $$, $$e ^\pm $$ $$\tau ^\mp $$ and $$\mu ^\mp $$ $$\tau ^\mp $$ could be mediated by a Higgs boson. At LEP2, limits on the cross-section of the $${e ^+e ^-} \rightarrow {e ^\pm } {\mu ^\mp } $$, $${e ^+e ^-} \rightarrow {e ^\pm } {\tau ^\mp } $$ and $${e ^+e ^-} \rightarrow {\mu ^\pm } {\tau ^\mp } $$ processes were obtained by the OPAL collaboration for centre-of-mass energies ($$\sqrt{s} $$) ranging from 192 to 209$$\,\mathrm {GeV}$$  [24]. These constraints can be translated into limits on the Higgs CLFV decay branching fraction [9, 25], which are on the order of $$10^{-8}$$ for a SM Higgs decay into an electron and muon [25]. Recent searches for the $$H\!\rightarrow {\mu ^\pm } {\tau ^\mp } $$ decay have been performed by the CMS  [26] and ATLAS  [27] collaborations for the Higgs boson with $$m_H$$ = 125$$\,{{\mathrm {GeV/}}c^2}$$. Upper limits on the branching fraction $${\mathcal {B}} {(H\!\rightarrow {\mu ^\pm } {\tau ^\mp } )}$$ have been placed by the two collaborations at 0.25% and 1.85%, respectively.

The possible existence of low-mass Higgs-like bosons is a feature of models like the two-Higgs-doublet models (2HDM) [28]. Searches for such particles have been performed by the ATLAS  [29] and CMS  [30] collaborations in the ditau decay mode. Another scenario is that of a hidden gauge sector [31, 32]. In this context, the BaBar and Belle collaborations have performed searches for a resonance with a mass below 10$\,{{\mathrm {GeV/}}c^2}$  [33, 34]. The LHCb collaboration has recently published the results of a search for dark photons decaying into the dimuon channel, placing a stringent limit for the production of a dimuon in the mass range from 10.6 to 70$\,{{\mathrm {GeV/}}c^2}$  [35].

The LHCb detector probes the forward rapidity region which is only partially covered by the other LHC experiments, and triggers on particles with low transverse momenta ($p_{\mathrm { T}}$), allowing the experiment to explore relatively small boson masses. In this paper a search for CLFV decays into a muon and a tau lepton of a Higgs-like boson with a mass ranging from 45 to 195$\,{{\mathrm {GeV/}}c^2}$ is presented, using proton-proton collision data collected at $\sqrt{s} =8$ $~{\mathrm {TeV}}$. The Higgs-like boson is assumed to be produced by gluon-fusion, similarly to the main production mechanism of the SM Higgs boson at LHC [36].^1^ The analysis is separated into four channels depending on the final state of the $\tau$ lepton decay: (i) single muon ${\tau ^-} \!\rightarrow {\mu ^-} {{\overline{\nu }} _\mu } {{\nu } _\tau }$, (ii) single electron ${\tau ^-} \!\rightarrow {e ^-} {{\overline{\nu }} _e} {{\nu } _\tau }$, (iii) single charged hadron ${\tau ^-} \!\rightarrow {{\pi } ^-} ({{\pi } ^0}){{\nu } _\tau }$, and (iv) three charged hadrons ${\tau ^-} \!\rightarrow {{\pi } ^-} {{\pi } ^-} {{\pi } ^+} ({{\pi } ^0}){{\nu } _\tau }$. They are denoted as $\tau _\mu$, $\tau _e$, $\tau _{h1}$, and $\tau _{h3}$ respectively. The main sources of background are ${Z} \!\rightarrow {\tau ^+\tau ^-}$ decays,^2^ heavy flavour production from QCD processes (“QCD” in the following) and electroweak boson production accompanied by jets (“$V\!j$ ”). This analysis utilizes reconstruction techniques and results obtained from the ${Z} \!\rightarrow {\tau ^+\tau ^-}$ measurement by the LHCb collaboration [37].

## Detector and simulation description

The LHCb detector [38, 39] is a single-arm forward spectrometer covering the $$2< \eta <5$$ pseudorapidity range, designed for the study of particles containing $$b $$ or $$c $$ quarks. The detector includes a high-precision tracking system consisting of a silicon-strip vertex detector surrounding the *pp* interaction region, a large-area silicon-strip detector located upstream of a dipole magnet with a bending power of $$4{\mathrm {\,Tm}}$$, and three stations of silicon-strip detectors and straw drift tubes placed downstream of the magnet. The tracking system provides a measurement of the momentum of charged particles with a relative uncertainty that varies from 0.5% at low momentum to 1.0% at 200$${\,\mathrm {GeV/}c}$$. The minimum distance of a track to a primary vertex (PV), the impact parameter (IP), is measured with a resolution of $$(15+29/p_{\mathrm { T}}){\,\upmu \mathrm {m}} $$, where $$p_{\mathrm { T}}$$ is the component of the momentum transverse to the beam, in $${\,\mathrm {GeV/}c}$$. Photons, electrons and hadrons are identified by a calorimeter system consisting of scintillating-pad (SPD) and preshower detectors (PS), an electromagnetic calorimeter (ECAL) and a hadronic calorimeter (HCAL). Muons are identified by a system composed of five stations of alternating layers of iron and multiwire proportional chambers.

Simulated data samples are used to calculate the efficiency for selecting signal processes, to estimate the residual background level, and to produce templates for the fit used to determine the signal yield. For this analysis, the simulation is validated primarily by comparing ${Z} \!\rightarrow l^+l^-$ decays in simulation and data. The Higgs boson is generated assuming a gluon-fusion process, and with mass values from 45 to 195$\,{{\mathrm {GeV/}}c^2}$ in steps of 10$\,{{\mathrm {GeV/}}c^2}$, using Pythia 8 [40, 41] with a specific LHCb configuration [42]. The parton density functions (PDF) are taken from the CTEQ6L set [43]. Decays of hadronic particles are described by EvtGen  [44], in which final-state radiation is generated using Photos  [45]. The interaction of the particles with the detector and its response are implemented using the Geant4 toolkit [46, 47] as described in Ref. [48]. Samples of $H\!\rightarrow {\mu ^\pm } {\tau ^\mp }$ decays generated at next-to-leading order precision by Powheg-Box [49–52] with the PDF set MMHT2014nlo68cl [53] are used for the signal acceptance determination.

## Signal selection

This analysis uses data corresponding to a total integrated luminosity of ${1976 \pm 23}\,\text{ pb }^{-1}$ [54]. The data collected uses a trigger system consisting of a hardware stage followed by a software stage. The hardware trigger requires a muon track identified by matching hits in the muon stations, as well as a global event cut (GEC) requiring the hit multiplicity in the SPD to be less than 600. The software trigger selects muons or electrons with a minimum $p_{\mathrm { T}}$ of 15${\,\mathrm {GeV/}c}$.

The $H\!\rightarrow {\mu ^\pm } {\tau ^\mp }$ candidates are identified and reconstructed into the four channels: ${\mu } \tau _e$, ${\mu } \tau _{h{1}}$, ${\mu } \tau _{h{3}}$ and ${\mu } \tau _\mu$. The $\tau _{h3}$ candidates are reconstructed from the combination of three charged hadrons from a secondary vertex (SV). The ${\mu } ^\pm \tau ^\mp$ candidates are required to be compatible with originating from a common PV. The muon track and the tracks used to reconstruct the tau candidate must be in the geometrical region $2.0<\eta <4.5$. Electron candidates are chosen amongst tracks failing the muon identification criteria and falling into the acceptance of the PS, ECAL, and HCAL sub-detectors. A large energy deposit, *E*, in the PS, ECAL, but not in HCAL is required, satisfying: $E_\text {PS} > 50\,\mathrm {MeV}$, $E_\text {ECAL}/p > 0.1$, and $E_\text {HCAL}/p < 0.05$, where $p$ is the reconstructed momentum of the electron candidate, after recovering the energy of the bremsstrahlung photons [55]. Charged hadrons are required to be in the HCAL acceptance, to deposit an energy $E_\text {HCAL}$ with $E_\text {HCAL}/p > 0.05$, and to fail the muon identification criteria. The pion mass is assigned to all charged hadrons.

The selection criteria need to be optimised over the $$m_H$$ range used in this analysis, from 45 to 195$$\,{{\mathrm {GeV/}}c^2}$$. Three different sets of selection criteria are considered, dubbed L-selection, C-selection, and H-selection. The C-selection is similar to that used for the analysis of $${Z} \!\rightarrow {\tau ^+\tau ^-} $$ decays [37]; as such, it is optimised for $${m_H} \sim m_Z$$. The L-selection and H-selection are optimised for the $$m_H$$ regions below and above the $$Z $$ mass respectively. All selection sets are applied in parallel to compute background estimation and exclusion limits. Subsequently, for each $$m_H$$ hypothesis, the chosen selection is that of L-, C-, or H-selection which provides the smallest expected signal limit, allowing precise separation between adjacent mass regions. As expected, it is found that the C-selection is optimal for a boson mass of 75 and 85$$\,{{\mathrm {GeV/}}c^2}$$. Below and above that range the best upper limits are obtained from the L- and H-selections, respectively. In the following discussion the requirements are applied identically for all decay channels and selection sets unless stated otherwise.

The tau candidates are selected with $p_{\mathrm { T}} >5{\,\mathrm {GeV/}c}$ for $\tau _e$,$\tau _\mu$, and $p_{\mathrm { T}} >10{\,\mathrm {GeV/}c}$ for $\tau _{h1}$. For the $\tau _{h3}$ candidate, the charged hadrons are required to have $p_{\mathrm { T}} > 1{\,\mathrm {GeV/}c}$ and one of them with $p_{\mathrm { T}} > 6{\,\mathrm {GeV/}c}$. They are combined to form the tau candidates, which are required to have $p_{\mathrm { T}} > 12{\,\mathrm {GeV/}c}$ and an invariant mass in the range 0.7 to 1.5$\,{{\mathrm {GeV/}}c^2}$. In the H-selection, the tau candidates must have $p_{\mathrm { T}}$ in excess of 20${\,\mathrm {GeV/}c}$. This requirement is not applied in the ${\mu } \tau _\mu$ channel as it favours the selection of ${Z} \!\rightarrow {\mu ^+\mu ^-}$ background. The muon from $H\!\rightarrow {\mu ^\pm } {\tau ^\mp }$ decay is expected to have a relatively large $p_{\mathrm { T}}$, thus the selection requires the muon $p_{\mathrm { T}}$ to be greater than 20${\,\mathrm {GeV/}c}$, 30${\,\mathrm {GeV/}c}$, and 40${\,\mathrm {GeV/}c}$ in the L-, C-, and H-selections, respectively. A tighter requirement of 50${\,\mathrm {GeV/}c}$ is applied for the muon in the ${\mu } \tau _\mu$ channel in the H-selection due to the ${Z} \!\rightarrow {\mu ^+\mu ^-}$ background. Additionally, for the ${\mu } \tau _e$ channel, the contribution from $W/Z\!\rightarrow {e} +\text {jet}$ background is suppressed by requiring the transverse momentum of the muon to be larger than that of the $\tau _e$ candidate.

The relatively large lifetime of the $\tau$ lepton is used to suppress prompt background. For the $\tau _{h3}$ candidate, a SV is reconstructed. A correction to the visible invariant mass, $m$, computed from the three-track combination, is obtained by exploiting the direction of flight defined from the PV to the SV. The relation used is $m_\text {corr} = \sqrt{m^2 + p^2 \sin ^2\theta } + p\sin \theta$, where $\theta$ is the angle between the momentum of the $\tau _{h3}$ candidate, and its flight direction. The $m_\text {corr}$ value is required to not exceed 3$\,{{\mathrm {GeV/}}c^2}$. A time-of-flight variable is also computed from the distance of flight and the partially reconstructed momentum of the $\tau$ lepton, and a minimum value of 30$\,\mathrm {fs}$ is required. The $m_\text {corr}$ and time-of-flight requirements together retain 80% of the signal, while rejecting about 75% of the QCD background. For tau decay channels with a single charged particle, it is not possible to reconstruct a SV, and a selection on the particle IP is applied. A threshold of IP $>10{\,\upmu \mathrm {m}}$ selects 85% of the $\tau _e$ and $\tau _{h1}$ candidates, and rejects about 50% of the $V\!j$ background. The threshold is increased to $50{\,\upmu \mathrm {m}}$ for $\tau _\mu$ candidates, in order to suppress ${Z} \!\rightarrow {\mu ^+\mu ^-}$ background. The prompt muon instead is selected by requiring IP less than 50${\,\upmu \mathrm {m}}$, allowing up to 50% rejection of QCD and ${Z} \!\rightarrow {\tau ^+\tau ^-}$ backgrounds.

The two leptons from the Higgs decay should be approximately back-to-back in the plane transverse to the beam. The absolute difference in azimuthal angle of muon and tau candidates is required to be greater than 2.7 radians. This rejects 50% of the $V\!j$ background. The transverse momentum asymmetry of the two particles, defined as $A_{p_\mathrm{T}} = {|p_{\mathrm { T}} {}_1 - p_{\mathrm { T}} {}_2|}/{(p_{\mathrm { T}} {}_1 + p_{\mathrm { T}} {}_2})$, can be used to effectively suppress various background processes. The background from the $V\!j$ processes is suppressed by up to 60% for the ${\mu } \tau _{h{1}}$ channel by requiring $A_{p_\mathrm{T}} < 0.4\;(0.5)$ in the L-selection (S-selection), because of the large $p_{\mathrm { T}}$ imbalance between the high-$p_{\mathrm { T}}$ muon from the vector boson and a hadron from a jet. For the ${\mu } \tau _e$ channel, the worse momentum resolution increases the average $A_{p_\mathrm{T}}$ value, hence a softer selection $A_{p_\mathrm{T}} < 0.6$ is used to preserve efficiency. On the contrary, for the ${\mu } \tau _\mu$ channel, a tighter cut is applied to suppress the dominant background from ${Z} \!\rightarrow {\mu ^+\mu ^-}$ decays. By requiring $A_{p_\mathrm{T}} > 0.3\ (0.4)$ in the L-selection and C-selection (H-selection), such background is reduced by 80%, while the signal decreases to 70%.

The two leptons from the Higgs decay are required to be isolated from other charged particles. Two particle-isolation variables are defined as $I_{p_\mathrm{T}} = (\vec {p}_\text {cone})_\text {T}$ and $\hat{I}_{p_\mathrm{T}} = {p_{\mathrm { T}}}/{(\vec {p} + \vec {p}_\text {cone})}_\text {T}$ where $\vec {p}$ is the momentum of the lepton candidate, the subscript $\text {T}$ denotes the component in the transverse plane, and $\vec {p}_\text {cone}$ is the sum of the momenta of all charged tracks within a distance $R_{\eta \phi }$ = 0.5 in the $(\eta ,\phi )$ plane around the lepton candidate. The isolation requirement $\hat{I}_{p_\mathrm{T}} >0.9$ is applied to the muon and tau candidates for all decay channels and selection sets, and retain 70% of the signal candidates while rejecting 90% of QCD events. In addition, a cut $I_{p_\mathrm{T}} < 2{\,\mathrm {GeV/}c}$ is applied in the L-selection to both candidates, as the lower $p_{\mathrm { T}}$ reduces the background rejection power of the $\hat{I}_{p_\mathrm{T}}$ variable.

The selection criteria common or specific to each selection set and decay channel are summarised in Table 1. The signal selection efficiencies are found to vary from 10 to 50%. Due to the kinematic selection, the decay channels are mutually exclusive and just one $\mu ^\pm$ $\tau ^\mp$ candidate per event is found.

**Table 1:** Requirements for each decay channel and selection set

Selection set	Variable	${\mu } \tau _e$	${\mu } \tau _{h{1}}$	${\mu } \tau _{h{3}}$	${\mu } \tau _\mu$
All	$p_{\mathrm { T}}$ ($\tau$) [${\,\mathrm {GeV/}c}$ ]	$>5$	$>10$	$>12$	$>5$
	$p_{\mathrm { T}} {(\tau _{h3}^\text {prong1})}$ [${\,\mathrm {GeV/}c}$ ]	–	–	$>1$	–
	$p_{\mathrm { T}} {(\tau _{h3}^\text {prong2})}$ [${\,\mathrm {GeV/}c}$ ]	–	–	$>1$	–
	$p_{\mathrm { T}} {(\tau _{h3}^\text {prong3})}$ [${\,\mathrm {GeV/}c}$ ]	–	–	$>6$	–
	$p_{\mathrm { T}} {(\mu )}-p_{\mathrm { T}} {(\tau )}$ [${\,\mathrm {GeV/}c}$ ]	$>0$	–	–	–
	$m$ ($\tau _{h3}$) [$\,{{\mathrm {GeV/}}c^2}$ ]	–	–	0.7–1.5	–
	$m_\text {corr}$ ($\tau _{h3}$) [$\,{{\mathrm {GeV/}}c^2}$ ]	–	–	$>3$	–
	Time-of-flight ($\tau _{h3}$) [$\,\mathrm {fs}$ ]	–	–	$>30$	–
	IP ($\tau$) [${\,\upmu \mathrm {m}}$ ]	$>10$	$>10$	–	$>50$
	IP ($\mu$) [${\,\upmu \mathrm {m}}$ ]	$<50$	$<50$	$<50$	$<50$
	$\Delta \phi$ [$\,\mathrm {rad}$ ]	$>2.7$	$>2.7$	$>2.7$	$>2.7$
	$\hat{I}_{p_\mathrm{T}}$ ($\tau$)	$>0.9$	$>0.9$	$>0.9$	$>0.9$
	$\hat{I}_{p_\mathrm{T}}$ ($\mu$)	$>0.9$	$>0.9$	$>0.9$	$>0.9$
L-selection	$p_{\mathrm { T}}$ ($\mu$) [${\,\mathrm {GeV/}c}$ ]	$>20$	$>20$	$>20$	$>20$
	$A_{p_\mathrm{T}}$	$<0.6$	$<0.4$	–	$>0.3$
	$I_{p_\mathrm{T}}$ ($\tau$) [${\,\mathrm {GeV/}c}$ ]	$<2$	$<2$	$<2$	$<2$
	$I_{p_\mathrm{T}}$ ($\mu$) [${\,\mathrm {GeV/}c}$ ]	$<2$	$<2$	$<2$	$<2$
C-selection	$p_{\mathrm { T}}$ ($\mu$) [${\,\mathrm {GeV/}c}$ ]	$>30$	$>30$	$>30$	$>30$
	$A_{p_\mathrm{T}}$	–	$<0.5$	–	$>0.3$
H-selection	$p_{\mathrm { T}}$ ($\tau$) [${\,\mathrm {GeV/}c}$ ]	$>20$	$>20$	$>20$	–
	$p_{\mathrm { T}}$ ($\mu$) [${\,\mathrm {GeV/}c}$ ]	$>40$	$>40$	$>40$	$>50$
	$A_{p_\mathrm{T}}$	–	–	–	$>0.4$

## Background estimation

**Fig. 1:**
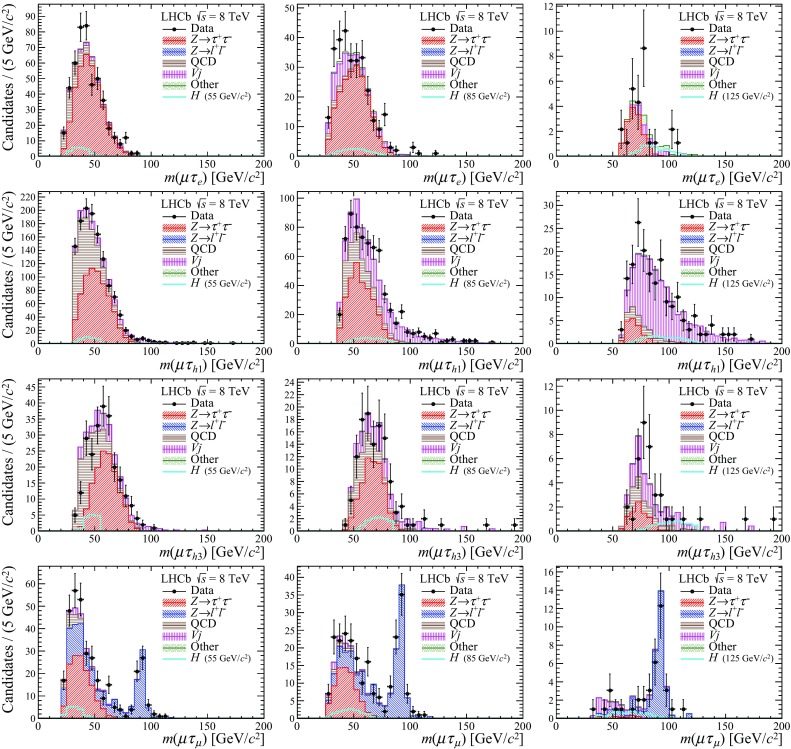
Invariant-mass distributions for the ${\mu } ^\pm \tau ^\mp$ candidates for the four decay channels (from top to bottom: ${\mu } \tau _e$, ${\mu } \tau _{h{1}}$, ${\mu } \tau _{h{3}}$, ${\mu } \tau _\mu$) and the three selections (from left to right: L-selection, C-selection, H-selection). The distribution of candidates observed (black points) is compared with backgrounds (filled colour, stacked), and with signal hypothesis (cyan). The signal is normalised to $\sqrt{N}$, with *N* the total number of candidates in the corresponding data histogram

Several background processes are considered: ${Z} \!\rightarrow {\tau ^+\tau ^-}$, ${Z} \!\rightarrow l^+l^-$ ($l={e},{\mu })$, QCD, $V\!j$, double bosons production ($VV$), ${t} {\overline{{t}}}$, and ${Z} \!\rightarrow {{b} {\overline{{b}}}}$. All backgrounds except ${Z} \!\rightarrow {\tau ^+\tau ^-}$ are estimated following the procedures described in Ref. [37]. The expected yields can be found in Table 2. The corresponding invariant-mass distributions compared with candidates observed in the data are shown in Fig. 1. For illustration, examples of $H\!\rightarrow {\mu ^\pm } {\tau ^\mp }$ distributions from simulation are also superimposed.

The $${Z} \!\rightarrow {\tau ^+\tau ^-} $$ background is estimated from the cross-section measured by the LHCb collaboration [37] where the reconstruction efficiency is determined from data, and the acceptance and selection efficiency are obtained from simulation. The estimated background includes a small amount of cross-feed from different final states of the tau decay, as determined from simulation. The $${Z} \!\rightarrow {\mu ^+\mu ^-} $$ background is dominant in the $${\mu } \tau _\mu $$ channel. The corresponding invariant-mass distribution is obtained from simulation and normalised to data in the $$Z $$ peak region, from 80 to 100$$\,{{\mathrm {GeV/}}c^2}$$. In order to suppress the potential presence of signal in this region, the muons are required to be promptly produced. For other channels, the $${Z} \!\rightarrow l^+l^-$$ decay becomes a background source in case a lepton is misidentified. This contribution is computed from the $${Z} \!\rightarrow l^+l^-$$ in data, and weighted by the particle misidentification probability obtained from simulation.

The QCD and $V\!j$ backgrounds are inferred from data using the same criteria as for the signal but selecting same-sign $\mu ^\pm$ $\tau ^\pm$ candidates. Their amounts are determined by a fit to the distribution of $p_{\mathrm { T}} {({\mu })}-p_{\mathrm { T}} {({\tau })}$, with templates representing each of them. The template for the QCD component is obtained from data requiring an anti-isolation $\hat{I}_{p_\mathrm{T}} < 0.6$ selection. The distribution obtained from simulation is used for the $V\!j$ component. Factors are subsequently applied for the correction of the relative yield of opposite-sign to same-sign candidates. For the QCD background the number of anti-isolated opposite-sign candidates found in data is used in the calculation of the correction factor, where it is found to be close to unity. The factors are found consistent with the simulation. The factors for the $V\!j$ component are taken from simulation, and are in general larger than unity (1.3 for ${\mu } \tau _e$ up to 3.1 for ${\mu } \tau _{h{1}}$, for the L-selection). The minor contributions from $VV$, ${t} {\overline{{t}}}$, and ${Z} \!\rightarrow {{b} {\overline{{b}}}}$ processes are estimated from simulation.

**Table 2:** Expected number of background candidates from each component, total background with uncertainty, and number of observed candidates with statistical uncertainty, from each decay channel and selection set

Selection set	Process	${\mu } \tau _e$	${\mu } \tau _{h{1}}$	${\mu } \tau _{h{3}}$	${\mu } \tau _\mu$
L-selection	${Z} \!\rightarrow {\tau ^+\tau ^-}$	$371.1\,\pm \,26.0$	$681.7 \,\pm \,47.1$	$135.1\,\pm \,11.7$	$137.4 \,\pm \,9.5$
	${Z} \!\rightarrow l^+l^-$	$8.2 \,\pm \,1.6$	$4.0\,\pm \,1.8$	–	$155.3 \,\pm \,5.0$
	QCD	$67.5 \,\pm \, 10.6$	$463.6 \,\pm \,5.4$	$93.1 \,\pm \, 10.9$	$19.4 \,\pm \,5.5$
	$V\!j$	$14.5 \,\pm \, 10.3$	$143.2 \,\pm \,58.6$	$40.1 \,\pm \, 15.8$	$10.7 \,\pm \,5.8$
	VV	$3.4 \,\pm \,0.3$	$0.9 \,\pm \,0.2$	$0.3 \,\pm \,0.1$	$0.3 \,\pm \,0.1$
	${t} {\overline{{t}}}$	$1.7 \,\pm \,0.1$	$1.3 \,\pm \,0.1$	$0.7\,\pm \,0.1$	$1.3 \,\pm \,0.2$
	${Z} \!\rightarrow {{b} {\overline{{b}}}}$	$0.2\,\pm \,0.2$	$0.2\,\pm \,0.2$	$0.1\,\pm \,0.1$	$0.2\,\pm \,0.2$
	Total background	$466.6\,\pm \,28.0$	$1294.9\,\pm \,75.5$	$269.4\,\pm \,20.3$	$324.5\,\pm \,12.5$
	Observed	$472.0\,\pm \,21.7$	$1284.0\,\pm \,35.8$	$240.0\,\pm \,15.5$	$344.0\,\pm \,18.5$
C-selection	${Z} \!\rightarrow {\tau ^+\tau ^-}$	$200.0\,\pm \,14.3$	$288.1\,\pm \,20.2$	$61.3\,\pm \,5.5$	$71.7\,\pm \,5.2$
	${Z} \!\rightarrow l^+l^-$	$8.0\,\pm \,1.7$	$4.3\,\pm \,1.8$	–	$126.7\,\pm \,4.5$
	QCD	$10.0\,\pm \,14.0$	$137.9\,\pm \,14.0$	$29.9\,\pm \,9.0$	$6.1\,\pm \,3.6$
	$V\!j$	$48.3\,\pm \,17.2$	$242.9\,\pm \,25.3$	$30.8\,\pm \,17.6$	$7.9\,\pm \,4.7$
	VV	$3.4\,\pm \,0.3$	$1.5\,\pm \,0.2$	$0.3\,\pm \,0.1$	$0.3\,\pm \,0.1$
	${t} {\overline{{t}}}$	$2.5\,\pm \,0.1$	$1.6\,\pm \,0.1$	$0.7\,\pm \,0.1$	$1.5\,\pm \,0.2$
	${Z} \!\rightarrow {{b} {\overline{{b}}}}$	$0.1\,\pm \,0.1$	$0.1\,\pm \,0.1$	$0.1\,\pm \,0.1$	$0.1\,\pm \,0.1$
	Total background	$272.3\,\pm \,17.8$	$676.4\,\pm \,35.2$	$123.1\,\pm \,15.0$	$214.3\,\pm \,8.1$
	Observed	$296.0\,\pm \,17.2$	$679.0\,\pm \,26.1$	$123.0\,\pm \,11.1$	$235.0\,\pm \,15.3$
H-selection	${Z} \!\rightarrow {\tau ^+\tau ^-}$	$13.7\,\pm \,1.8$	$18.4\,\pm \,1.6$	$8.9\,\pm \,1.1$	$2.2\,\pm \,0.4$
	${Z} \!\rightarrow l^+l^-$	$4.7\,\pm \,1.1$	$2.5\,\pm \,1.1$	–	$33.7\,\pm \,2.3$
	QCD	–	$15.8\,\pm \,6.3$	$9.7\,\pm \,5.1$	–
	$V\!j$	$3.5\,\pm \, 2.6$	$142.6 \,\pm \, 26.0$	$18.6\,\pm \, 16.5$	$7.8 \,\pm \, 4.0$
	VV	$1.7 \,\pm \, 0.2$	$1.0 \,\pm \, 0.2$	$0.1 \,\pm \, 0.1$	$0.2 \,\pm \, 0.1$
	${t} {\overline{{t}}}$	$1.2 \,\pm \, 0.1$	$0.9 \,\pm \, 0.1$	$0.4 \,\pm \, 0.1$	$0.8 \,\pm \, 0.1$
	${Z} \!\rightarrow {{b} {\overline{{b}}}}$	$0.1 \,\pm \, 0.1$	$0.1 \,\pm \,0.1$	$0.1 \,\pm \,0.1$	$0.1 \,\pm \,0.1$
	Total background	$24.9 \,\pm \,3.4$	$181.2 \,\pm \, 26.7$	$37.8 \,\pm \, 13.6$	$44.7 \,\pm \, 4.6$
	Observed	$27.0 \,\pm \,5.2$	$184.0 \,\pm \,13.6$	$37.0 \,\pm \,6.1$	$39.0 \,\pm \,6.2$

## Results

The signal cross-section multiplied by the branching fraction is given by

1 $$\begin{aligned} \sigma (gg\!\rightarrow H\!\rightarrow {\mu ^\pm } {\tau ^\mp } ) = N_\text {sig}/ (\mathcal {L} \cdot {\mathcal {B}} {({\tau } \!\rightarrow X)} \cdot \varepsilon ), \end{aligned}$$

where $N_\text {sig}$ is the signal yield obtained from the fit procedure described below, $\mathcal {L}$ the total integrated luminosity, ${\mathcal {B}} {({\tau } \!\rightarrow X)}$ the tau branching fraction, and $\varepsilon$ the detection efficiency. The latter is the product of acceptance, reconstruction, and offline selection efficiencies. These efficiencies are obtained from simulated samples and data for each decay channel and selection set, following the methods developed for the ${Z} \!\rightarrow {\tau ^+\tau ^-}$ measurement [37]. The acceptance obtained from the Powheg-Box generator is identical for the ${\mu } \tau _e$, ${\mu } \tau _{h{3}}$, and ${\mu } \tau _\mu$ channels, varying from 1.0% for ${m_H} = 195\,{{\mathrm {GeV/}}c^2}$ to 3.2% for ${m_H} = 75\,{{\mathrm {GeV/}}c^2}$. The reconstruction efficiency, which is the product of contributions from trigger, tracking, and particle identification, is in the range 40–70%, but only about 15% in the case of the ${\mu } \tau _{h{3}}$ channel because of the limited tracking efficiency for the low-momentum hadrons. With the exception of the ${\mu } \tau _\mu$ channel, the selection efficiency is 18–30% in the L-selection, and 24–49% in the C-selection and H-selection. In the case of the ${\mu } \tau _\mu$ channel, the tighter selection on the muon $p_{\mathrm { T}}$ and impact parameter reduces the selection efficiency to 10–15%.

The systematic uncertainties are summarised in Table 3. The uncertainty on the acceptance receives contributions from the gluon PDF uncertainty, as well as from factorization and renormalisation scales. The uncertainties on the reconstruction and selection efficiencies are estimated from simulation and are calibrated using data as described in Ref. [37]. The uncertainty associated with the invariant-mass shape is handled by selecting the weakest expected limits among the different choices of distribution (kernel estimation and histograms with different bin widths are used). The uncertainties on the integrated luminosity and acceptance are fully correlated among channels, while only a partial correlation is found for the reconstruction efficiency uncertainties. All the other uncertainties are taken as uncorrelated.

**Table 3:** Relative systematic uncertainties (in %) on the normalisation factors in the cross-section calculation. When the uncertainty depends on $m_H$ a range is indicated

	${\mu } \tau _e$	${\mu } \tau _{h{1}}$	${\mu } \tau _{h{3}}$	${\mu } \tau _\mu$
Luminosity	1.16	1.16	1.16	1.16
Tau branching fraction	0.22	0.18	0.48	0.23
PDF	2.6–7.1	3.5–7.2	2.6–7.3	3.0–7.9
Scales	0.9–1.9	0.8–1.7	0.9–1.7	0.9–1.9
Reconstruction efficiency	1.8–3.6	1.9–5.4	3.3–7.1	1.5–3.3
Selection efficiency	2.5–6.0	1.9–4.1	4.0–9.3	3.8–8.5

The signal yield is determined from a simultaneous extended likelihood fit of the binned invariant-mass distributions of the $\mu$ $\tau$ candidates. The distributions for signal are obtained from simulation, while distributions of the different background sources are obtained using the method described in Sect. 4. The amount of each background component as well as other terms in Eq. (1) containing uncertainties are treated as nuisance parameters and are constrained to a Gaussian distribution with mean and standard deviation corresponding to the expected value and its uncertainty, respectively.

**Fig. 2:**
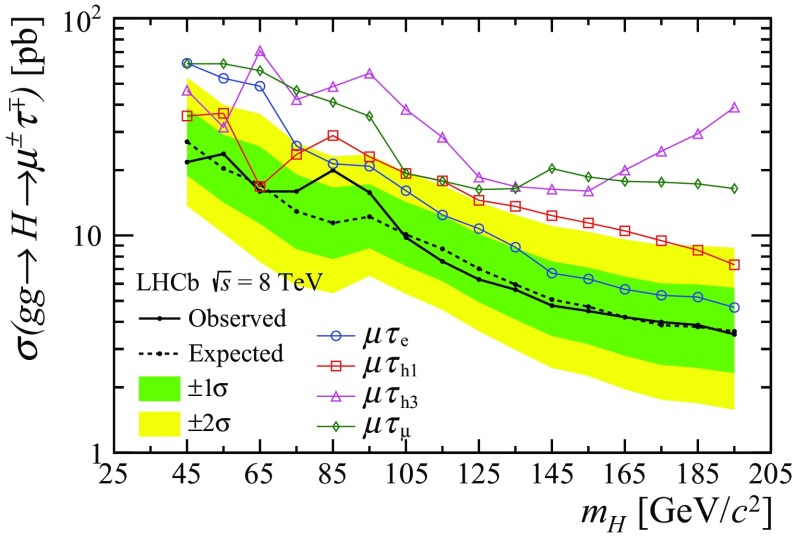
Cross-section times branching fraction 95% CL limits for the $H\!\rightarrow {\mu ^\pm } {\tau ^\mp }$ decay as a function of $m_H$, from the simultaneous fit. The observed limits from individual channels are also shown

The fit results for all $m_H$ values are compatible with a null signal, hence cross-section upper limits are computed. The exclusion limits of $\sigma (gg\!\rightarrow H\!\rightarrow {\mu ^\pm } {\tau ^\mp } )$ defined at 95% confidence level are obtained from the $\text {CL}_\text {s}$ method [56]. As mentioned before, for each mass hypothesis the selection considered is that providing the smallest expected limit. The $\sigma (gg\!\rightarrow H\!\rightarrow {\mu ^\pm } {\tau ^\mp } )$ exclusion limits are shown in Fig. 2, ranging from 22$\,{\mathrm {pb}}$ for $m_H$ = 45$\,{{\mathrm {GeV/}}c^2}$ to 4$\,{\mathrm {pb}}$ for $m_H$ = 195$\,{{\mathrm {GeV/}}c^2}$. In the particular case of $m_H$ = 125$\,{{\mathrm {GeV/}}c^2}$, using the production cross-section from Ref. [57] gives a best fit for the branching fraction of ${\mathcal {B}} {(H\!\rightarrow {\mu ^\pm } {\tau ^\mp } )} = -\,2^{+14}_{-12}\%$ and an observed exclusion limit ${\mathcal {B}} {(H\!\rightarrow {\mu ^\pm } {\tau ^\mp } )} < 26\%$. The corresponding exclusion limit on the Yukawa coupling is $\sqrt{|Y_{\mu \tau }|^2 + |Y_{\tau \mu }|^2} < 1.7\times 10^{-2}$, assuming the decay width $\Gamma _\text {SM} = 4.1{\,\mathrm {MeV/}c^2}$ [58].

## Conclusion

A search for Higgs-like bosons decaying via a lepton-flavour-violating process $H\!\rightarrow {\mu ^\pm } {\tau ^\mp }$ in *pp* collisions at $\sqrt{s}$ = 8$~{\mathrm {TeV}}$ is presented, with the tau lepton reconstructed in leptonic and hadronic decay modes. No signal has been found. The upper bound on the cross-section multiplied by the branching fraction, at 95% confidence level, ranges from 22$\,{\mathrm {pb}}$ for a boson mass of 45$\,{{\mathrm {GeV/}}c^2}$, to 4$\,{\mathrm {pb}}$ for 195$\,{{\mathrm {GeV/}}c^2}$. The search provides information complementary to the ATLAS and CMS collaborations.

## Footnotes

1 The remaining Higgs production modes (e.g., $\sim 10\%$ from Vector-Boson Fusion) are neglected in this study.

2 Throughout this note, $$Z $$ implies $${Z}/\gamma ^*$$, i.e. includes contributions from $$Z $$ boson production, virtual photon production, and also their interference.
